# Cognition, emotion, and Obstructive Sleep Apnoea Syndrome before and after severe weight loss treatment

**DOI:** 10.5935/1984-0063.20210009

**Published:** 2022

**Authors:** Olga Rodrigues Ribeiro, Paula Costa, Isabel do Carmo, Teresa Paiva, Maria Luísa Figueira

**Affiliations:** 1 Hospital de Egas Moniz, Neuropsychology Unit; Instituto de Saúde Ambiental - Lisboa - Lisboa - Portugal.; 2 IPSS, Associação Living Care - Madeira - Madeira - Portugal.; 3 Hospital de Santa Maria, Endocrinology, Diabetes and Metabolism Department - Lisboa - Lisboa - Portugal.; 4 CENC, Sleep Medicine Centre - Lisboa - Lisboa - Portugal.; 5 Hospital de Santa Maria, Psychiatry Department - Lisboa - Lisboa - Portugal.

**Keywords:** Cognition, Expressed Emotion, Sleep Apnea Syndromes, Weight Reduction Programs

## Abstract

**Introduction:**

This study aimed to compare the results of a conservative method and the Sleeve Gastrectomy procedure for weight loss on the cognitive-emotional performance of severely obese women assessed for Obstructive Sleep Apnoea Syndrome.

**Material and methods:**

Two samples consisting of females, approved for Sleeve Gastrectomy (n = 21) and Conservative Treatment (n = 21) underwent night polysomnography and completed a battery of neuropsychological and emotional tests before and 6 months after the interventions. We compared intra- and inter-sample results, post interventions result to controls, and treated patients with Obstructive Sleep Apnoea Syndrome.

**Results:**

Anthropometry, immediate memory, attention, executive functions, and emotional maladjustment improved after the interventions. The conservative method showed better results for inhibitory control, and surgery showed better results for cognitive flexibility, speed of information processing and general cognitive and emotional performance for women with Obstructive Sleep Apnoea Syndrome. Learning decreased following both interventions. Memory and cognitive flexibility were lower in the comparison group than in control groups.

**Discussion:**

Treatments impacted different cognitive domains with probable influence on the objectives achieved. Lower middle pressure for behaviour modification may have reduced learning after interventions. The reduction of depression/anxiety in women with Obstructive Sleep Apnoea may result from the improvement of the social effects of both conditions. Although with better results for the surgical method, anthropometric reductions in both methods, positively influenced the cognitive/emotional domains. The maintenance of cognitive weaknesses implies longer and more focused interventions to avoid the regression of results like the worsening of Obstructive Sleep Apnoea Syndrome.

## INTRODUCTION

Obesity has been increasing in several regions of the world, including Europe. A recent study showed an average prevalence across EU member states in 2014 of 15.9%. It was estimated that the obesity pandemic will, on average for the 18 European countries studied, reach its peak in 2037 with a prevalence level of 31% for those aged between 20 and 84^1^.

Body mass index (BMI) is the most common measurement of obesity. An increased BMI (>30kg/m^2^) is a marker for a large number of chronic diseases, such as cardiovascular diseases, diabetes mellitus (DM), obstructive sleep apnoea syndrome (OSA), depressive syndrome, and many cancers. Obesity also has deleterious effects in the cognitive and emotional domains. These effects worsen with age and are associated with comorbidities (e.g., hypertension and dyslipidaemia), low cognitive reserve (CR), and severe obesity (BMI≥40kg/m^2^)^2^.

In fact, structural changes in brain volume and the frontal and temporal cortex are inversely associated with BMI. These changes mostly result from impairments of a vascular nature that are connected to decreases in cognitive control and executive functioning as well as affective states such as anxiety and depression^3,4^. CR, which reflects the mind’s ability to optimise performance in the face of brain pathology through differential recruitment of brain networks, is thought to have a protective effect on BMI and cognitive performance^5,6^. This protective effect was shown for domains that often exhibit deficits in obese persons, such as attention, executive function (EF), and memory, suggesting that CR attenuates their expression, especially in midlife^7^.

OSA is the most common sleep disorder. It is a chronic condition, the gold-standard evaluation of which is the nocturnal polysomnography (PSG) and it is nearly twice as common in obese adults as it is in adults of normal weight^8^. OSA affects mental health and is considered a major contributor to cerebrovascular changes such as structural and functional deterioration that lead to cognitive impairments and neurodegeneration^9^. Sufficient weight loss can confer benefits and significantly mitigate symptoms of OSA (e.g., snoring, insomnia, sleepiness, fatigue, and poor concentration). Permanent weight reduction through bariatric surgery (BS) is the most effective method for achieving these benefits, with a mitigating impact on OSA, reported in 86% of BS patients who experienced OSA prior to surgery^10^.

Obese patients with OSA should be provided with additional information about the benefits associated with dietary and surgical weight reduction on cognitive results. This is particularly the case for those that do not tolerate treatment with continuous positive air pressure (CPAP), patients that refuse surgical treatments or patients suffering from a psychiatric disease that need to be stabilised.

Even in small amounts, weight reduction should always be favoured. However, the efficacy of conservative treatment interventions (CTs) is controversial because of the inconsistent and variable long-term sustainability of the resulting weight loss. BS is the most significant treatment available for obesity and obesity-related comorbidities like OSA, including for the amelioration of abnormalities in brain regions and cognitive impairments as early as two weeks after the intervention^11^. The present work was based on the near absence of longitudinal studies comparing the results of a CT versus BS weight loss in relation to the cognitive and emotional performance of severely obese women who underwent a PSG for OSA diagnosis and were between 18 and 65 (and thus considered not yet elderly)^12^.

One portion of the sample group opted for sleeve gastrectomy (SG), a form of BS, performed under the guidance of a multidisciplinary team. The other portion chose a CT of a structured nutritional weight loss approach. Six months after SG and following an equal horizon time for CT: 1) results were compared within and between the groups, 2) were compared respectively against controls, and 3) were compared for women of both groups with the diagnosis of OSA. It was hypothesised that diet and bariatric patients would exhibit an improvement in cognitive and emotional performance in different domains.

It was predicted that women with OSA and SG would present better results than women with OSA performing CT. This was expected once SG was associated with a faster and sustainable reduction on anthropometric measurements that is directly associated with a significant increase of cognitive and emotional OSA domains. Independently of the methods employed, it was predicted that both samples of those with OSA would present an improved cognitive profile.

## MATERIAL AND METHODS

### Sample

The present study is part of longitudinal research presented to the ethics committee of the main city hospital in Lisbon. It was approved in March 2012 and took place between May 2012 and July 2016.

Those eligible for this study were persons aged 18 to 65 who had no diagnosis of psychiatric or neurological disorders. Eligible participants had at least four years of basic schooling, corrected hearing and vision and have been selected for SG or CT by the team at the morbid obesity consultation centre of the referred hospital.

We followed 49 patients (42 women and 7 men) throughout the period of their respective weight loss interventions. The patients belonged to an initial group of 120 that performed a neuropsychological and emotional evaluation, of which 61 (49 women and 12 men) went on to undergo a PSG in the pulmonology department^2,13^. PSGs were performed with an *Alice* 5 device (Philips Respironics^®^, USA), and were reviewed by a pulmonologist with training in sleep disorders according to internationally agreed criteria established by the American Academy of Sleep Medicine^14^.

Patients experienced the full extent of the scheduled nutritional and psychological structured intervention implemented at the centre^15-17^. Because of a substantial dropout rate and the sample size of men being so small, men’s results were omitted from the final study. We compared 21 women who had SG with 21 that followed a CT approach. This comparison was conducted within and between the groups six months after the respective treatments.

### Material and Methods

A sociodemographic and clinical questionnaire was administered to collect personal and social characteristics and relevant clinical data. A paper-and-pencil neuropsychological battery of evaluations was applied, which comprised, the Digit Span, Digit Symbol, Search Symbol and Vocabulary tests of the Wechsler Adult Intelligence Scale, third edition (WAIS-III)^18^; the Rey-Osterrieth Complex Figure Test, copy and memory (RCF)^19^; the Rey Auditory Verbal Learning Test (RAVLT)^20^; the Stroop Colour and Word Test^21^; the Trail Making Test, form A and B (TMT)^20^; the Wisconsin Card Sorting Test (WCST)^22^; and the Portuguese version of the Hopkins Symptom Checklist 90-Revised (SCL-90-R)^23^.

The tests in the neuropsychological battery were chosen with an acknowledgement of the impact of obesity on cognitive performance^2,24^. The results of this test battery provided a baseline for measuring attention and working memory, fine motor control and learning speed, processing speed, level of education, perceptual/visuospatial ability and memory, episodic memory, resistance to interference, cognitive flexibility, problem-solving and abstract thinking and distress symptomatology.

### Procedures

At the end of their first required endocrinology consultation, patients were invited to participate in the study. We explained the purpose of the study to each patient, confirmed their voluntary interest and obtained informed consent information. We accessed data from patients’ clinical records on vascular risk factors such as hypertension (HTN), DM and dyslipidaemia, and categorised each as a dichotomous variable that, together with lifestyle habits (tobacco and alcoholic beverage consumption, physical activity) was ‘present’ or ‘not present’.

We compared anthropometric, cognitive, and emotional results of: 1) 21 women who followed a CT approach and 21 women that had an SG, within and between the groups six months after the respective treatments; 2) both groups after treatments with the respective control groups; and 3) 14 women from the CT group and 13 women from the SG group that had OSA, within and between the groups six months after the respective treatments.

For each evaluation, we measured weight, height, neck circumference (NC), waist circumference (WC), and hip circumference (HC). We calculated BMI as the weight (kg) divided by the height (m^2^), the waist-to-hip ratio (WHR) as the WC in cm divided by the HC in cm, and the waist-to-height ratio (WHTR) as the WC in cm divided by the height in cm. We calculated the mean percentage of weight loss after both interventions (%WL).

Patients with an apnoea-hypopnoea index (AHI)<5 were considered without OSA, and those with an AHI≥5 had been diagnosed with OSA^14^. OSA was categorised as a dichotomous variable: ‘present’ or ‘not present’. Years of schooling served as a proxy for estimated CR. Values greater than the median of 4 were scored as ‘high’ CR and values equal to, or less than, 4 were scored as ‘low’ CR^5^.

Nutritionists at the morbid obesity consultation centre had scheduled a consultation with each patient every two months. The psychologists’ protocol included five sessions of psychological evaluation and a psychological counselling session once every three months^15-17^. Neuropsychological assessments were carried out individually, between 2 and 6 p.m., had an average duration of 45 minutes and were conducted by a psychologist with a specialisation in neuropsychology.

### Statistical analysis

Statistical analysis was performed with IBM SPSS Statistics for Windows, Version 24.0 (IBM Corp., Armonk, NY, U.S.). Since most of the variables did not follow a normal distribution and the sample sizes were both less than 30, we used non-parametric analytic methods: Spearman’s correlation test for quantitative measurements, Kendall’s Tau_b for correlations between quantitative and nominal measures, Chi-Square for dummy variables, Man-Whitney test to compare independent samples, and Wilcoxon test to compare measurements before and after treatment. The statistical significance level was set at *p*<.05.

## RESULTS

The age of the 42 women in the clinical sample ranged between 21 and 59 years (40.95y±11.87). The sociodemographic and clinic characteristics are set out in Table 1, and the anthropometric measurements of both clinical and control groups are presented in Table 2.

**Table 1:** Characterization of the clinical sample (n=42).

	n	%
**Age groups**		
20-30 years old	11	26.2
31-40 years old	11	26.2
41-50 years old	7	16.7
>50 years old	13	31.0
**Marital status**		
Singles	5	11.9
Married/unmarried couples	30	71.4
Separated/divorced	7	16.7
**Employment status**		
Employed	20	47.6
Unemployed	18	42.9
Pensioners	3	7.1
Others	1	2.4
**Qualifications**		
1º Cycle of basic school	6	14.3
2º Cycle of Basic school	5	11.9
3º Cycle of basic school	13	31.0
Secondary school	14	33.3
Bachelor/graduation/master	4	9.5
**Income**		
Without income	4	9.5
<500 euros	19	45.2
500-750 euros	14	33.3
750-1000 euros	2	4.8
1000-1500 euros	1	2.4
>1500 euros	2	4.8
**OSA diagnosis**		
Without OSA (AHI)<5)	15	35.7
With diagnosed OSA (AHI≥5)	27	64.3
**Clinical diagnosis**		
Arterial hypertension	22	52.4
Type 2 diabetes	8	19
Dislipidaemia	4	9.5
**Lifestyle habits**		
Tobacco consumption	8	19
Alcoholic beverages consumption	5	11.9
Regular physical activity	10	23.8
**Obesity course**		
Overweight since childhood	19	45.2
Expert-oriented weight loss trails	40	95.2
Assignment of weight to wrong daily habits	18	42.9
Assignment of weight to emotional causes	20	47.6
**Cognitive reserve**		
Low cognitive reserve	24	57.1
High cognitive reserve	18	42.9

* *p*<0.05;

** *p*<0.01; SD = Standard deviation.

**Table 2:** Descriptive statistics of the anthropometric measurements of the clinical sample and controls.

Anthropometric measurements	Clinical sample (n=42)	Control sample (n=40)
Weigh (kg)	123.13±17.49	55.66±7.61
Height (cm)	1.60±0.69	1.63±0.77
Neck circumference (cm)	42.24±5.30	31.58±2.13
Waist circumference (cm)	127.59±13.23	78.15±9.16
Hip circumference (cm)	138.92±9.88	94.66±8.02
Hip waist ratio	0.92±0.08	0.82±0.06
Waist height ratio	0.79±0.09	0.47±0.06
BMI (kg/m^2^)	47.76±6.75	21.64±2.30

**Cognitive reserve:** of the total patients, 57.1% (n=24) had a low CR, and 42.9% (n=18) had a high CR. The youngest group (19.2% with low CR vs. 81.8% with high CR) and the oldest group (92.3% with low CR vs. 7.7% with high CR) had the most patients with the high and the low CR, respectively. The low CR group had the most patients with HTN (*χ^2^*=4.582, *p*=.032) and who refered not to practice any kind of physical activity (*χ^2^*=7.394, *p*=.007). The significant associations between CR, HTN, DM, and OSA with sociodemographic, anthropometric, and cognitive measurements can be seen in Table 3.

**Table 3:** Significant non-parametric correlations (Spearmen, Tau_b Kendall, and Qui-Square) between sociodemographic, anthropometric, cognitive measurements, and CR, HTN, diabetes, and dyslipidemia of the clinical sample before the treatment (n=42).

Variables	Cognitive reserve	HTN	Diabetes	OSA presence
**Sociodemographic**				
Age	-0.450**	0.350*		
**Anthropometric measurements**				
Weight			0.301*	
Neck circumference	-0.351**	0.286*	0.340*	
Waist circumference			0.377**	
Hip circumference			0.350*	
Waist hip ratio			0.290*	
Waist height ratio	-0.255*		0.391**	
BMI			0.351**	
**Cognitive measurements**				
Immediate recall	0.327*	-0.331*		
Retention index		-0.325*		
Digit symbol	0.463**	-0.307*		
Search symbol	0.375**	-0.318*		
Digit span		-0,322*		
TMT A (time)	-0.297*			
TMT B (time)	-0.350**	0.280*		
WCST failure to maintain set			0.296*	
**Risk factors**				
HTN	4.582*			6.185*
DM				5.490*
**Lifestyle habits**				
Regular physical activity	7.394**			

* *p*<0.05;

** *p*<0.01.

**Obstructive sleep apnoea:** of patients from the CT group, 51.85% (n=14) had an OSA diagnosis compared to 48.15% (n=13) of those from the BS group.

**Dropout:** the interval between the first and the last neuropsychological evaluation had a mean time of 24.97months±7.65; the attrition rate was 40.83%.

**Percentage of weight lost:** the mean %WL for the clinical sample was 14.64 ±15.25. For the CT group the mean %WL was 3.72 ±8.80, for the SG group was 25.55 ±12.22 and significantly higher for those with OSA undergoing SG than for those following the CT (*U*=-3.931, *p*=.000). For the clinical sample, we found a negative association between %WL and the distress symptoms at the final of treatments: global severity index (*r*=-.453, *p*=.003), positive symptom distress index (*r*=-.422, *p*=.005), somatisation (*r*=-.498, *p*=.001), obsessive-compulsive (*r*=-.355, *p*=.021), interpersonal sensitivity (*r*=-.424, *p*=.005), depression (*r*=-.476, *p*=.001), anxiety (*r*=-.328; *p*=.034), paranoid ideation (*r*=-.321, *p*=.038), and psychoticism (*r*=-.377, *p*=.014).

**Comparison within and between clinical groups:** we did not find differences for age, qualifications, income employment, marital status, or risk factors when we compared the CT (n=21) and BS (n=21) groups. Anthropometric, neuropsychological, and emotional differences within groups and between groups after treatment are expressed in Table 4 and differences after treatment in CT (n=14), and SG (n=13) samples with OSA are shown in Table 5.

**Table 4:** Changes within and between clinical samples [means (standard deviations)] performed by Wilcoxon test and Man-Whitney test.

	Conservative Treatment (CT) (n=21) Mean (SD)		Differences of medians (p-value) before and after CT	Sleeve Gastrectomy (SG) (n=21) Mean (SD)		Differences of medians (p-value) before and after SG	Differences of medians between groups after treatment (p-value)
	**t0**	**t1**		**t0**	**t1**		
Anthropometric measurements							
Weight (Kg)	123.30 (17.14)	118.67 (19.06)	.126	122.97 (18.26)	91.70 (21.19)	.000**	.000**
Neck circumference (cm)	43.24 (6.79)	41.57 (4.51)	.065	41.23 (3.06)	37.28 (2.34)	.000**	.001**
Waist circumference (cm)	126.90 **(**15.28)	127.57 (15.03)	.825	128.28 (11.16)	108.52 (15.48)	.000**	.000**
Hip circumference (cm)	139.66 (9.73)	139.04 (11.46)	.975	138.19 (10.22)	122.90 (14.92)	.000**	.001**
BMI (Kg/m^2^)	47.80 **(**7.65)	46.34 (8.15)	.205	47.73 (5.91)	35.47 (7.80)	.000**	.000**
Waist-to-hip ratio (cm)	.91 (.10)	.91 (.64)	.717	.92 (.05)	.88 (.05)	.004**	.026*
Waist-to-height ratio (cm)	.79 **(**.11)	.79 (.10)	.90	.79 (.07)	.67 (.09)	.000**	.001**
**Cognitive measurements (raw data)**							
Immediate recall	42.60 (8.75)	49.90 (9.30)	.001**	43.05 (7.13)	47.19 (8.76)	.040*	.308
Learning	15.76 (5.64)	10.38 (6.85)	.022*	15.90 (5.53)	10.29 (6.61)	.002**	.930
Interference	-7.30 (7.44)	1.24 (7.35)	.001**	-4.96 (8.82)	-1.46 (8.20)	.149	.195
Digit symbol	59.24 (15.32)	66.14 (17.09)	.002**	60.71 (20.58)	68 (21.94)	.000**	.850
Search symbol	26.48 (7.58)	29.95 (8.06)	.006**	28.76 (9.43)	30.86 (9.76)	.051	.970
RCF copy	30.04 (4.17)	27.66 (3.66)	.005**	28.85 (4.86)	27.83 (5.17)	.140	.561
TMT A (time)	49.71 (18.90)	43.67 (19.78	.014*	45 (15.96)	38.52 (12.25)	.061	.326
TMTB (time)	114.43 (51.44)	107.90 (57.97)	.130	108.81 (55.52)	88.19 (29.90)	.008**	.497
WCST nr. administered trails	111.95 (20.70)	108.71 (24.78)	.600	114.90 (21.21)	103.76 (26.63)	.025*	.531
WCST % perseverative responses	21.45 (11.15)	20.75 (12.88)	.590	27.77 (18.06)	19.89 (17.80)	.021*	.488
WCST % perseverative errors	19.20 (9.61)	18.56 (10.44)	.664	23.50 (13.74)	17.53 (13.79)	.027*	.521
WCST % conceptual level responses	54.76 (22.07)	54.63 (26.55)	.376	45.57 (25.98)	56.33 (29.72)	.030*	.660
Emotional measurements (raw data)							
Global severity index	1.22 (.48)	1.12 (.60)	.262	1.19 (.62)	.59 (.36)	.000**	.003**
Positive symptom distress index	1.90 (.38)	1.69 (.41)	.027*	1.69 (.44)	1.30 (.18)	.000**	.000**
Somatization	18 (5.41)	15.81 (7.18)	.343	16.81 (9.02)	8.76 (4.14)	.001**	.001**
Obsessive-compulsive	14.71 (6.80)	13.71 (8.03)	.443	15.38 (7.24)	8.71 (4.16)	.000**	.041*
Interpersonal sensitivity	13.29 (7.32)	11.43 (8.55)	.382	13.33 (8.18)	6.05 (4.79)	.000**	.039*
Depression	19.62 (8.51)	17.76 (9.48)	.210	17.57 (9.97)	8.62 (5.58)	.000**	.002**
Anxiety	10.57 (6.31)	9.10 (6.46)	.020*	10.19 (7.10)	4.71 (4.30)	.001**	.020*
Hostility	6.62 (4.68)	4.86 (3.82)	.004**	5.95 (4.23)	2.90 (2.68)	.002**	.115
Phobic anxiety	4.33 (4.12)	4.38 (4.16)	.954	5.38 (5.18)	1.81 (2.67)	.005**	.019*
Paranoid ideation	6.48 (3.82)	7.10 (4.48)	.022*	6.95 (3.76)	4.05 (3.07)	.001**	.026*
Psychoticism	6.71 (5.64)	6.76 (6.12)	.849	6.81 (5.28)	3.10 (3.19)	.002**	.041*

* *p*<0.05;

** *p*<0.01.

**Table 5:** Changes within and between the clinical samples with OSA performed by Wilcoxon test and Man-Whitney test, respectively.

Variables	Differences of medians for CT sample with OSA before and after treatment (n=14)	Differences of medians for SG with OSA before and after treatment (n=13)	Differences of medians between samples after treatment (p-value)
**Anthropometric measurements**			
Weigh	-2.009*	-3.180**	.005**
Neck circumference		-3.068**	.002**
Waist circumference		-3.183**	.002**
Hip circumference		-2.713**	
Waist-to-height ratio		-3.180**	.002**
Body mass index		-3.180**	.002**
**Cognitive measurements**			
Immediate recall	-2.413*		
Learning		-2.200*	
Digit symbol	-2.451*	-2.982**	
Search symbol		-3.183**	
Digit span		-3.204**	
Interference	-2.982*		.025*
Trail making test A (time)	-2.344*		
Trail making test B (time)		-2.551*	
Rey complex figure (copy)		-1.967*	
**Emotional measurements**			
Global severity index	-2.354*	-3.040**	
Positive symptom distress index	-2.386*	-2.667**	.043*
Somatization	-2.477*	-2.171*	
Obsessive-compulsive		-2.907**	
Interpersonal sensitivity		-3.185**	
Depression	-2.232*	-3.181**	.038*
Anxiety	-2.302*	-2.538*	
Hostility	-2.501*	-2.358*	
Phobic anxiety			
Paranoid ideation		-2.827**	
Psychoticism		-2.536**	

**Comparison between controls, CT, and SG, respectively:** the results of cognitive and emotional performance were compared with the results of a sample of 40 women (20 for each group respectively), controlled for age and schooling. The mean age of the control groups was (41.98y±11.87) without significant differences between the CT (41y±10.65) and SG (40.90±13.24) groups. The CT group results were lower than those of the control group for immediate recall (*W*=-3.202, *p*=.001), TMT form B (*W*=-1.979, *p*=.048), RCF copy (*W*=-2.675, *p*=.007), and somatisation (*W*=-2.615, *p*=.009). The SG group presented a higher percentage of perseverative responses (W =-2.596, p =009), and perseverative errors of the WCST (W =-2.395, p = .017) than the control group.

**Comparison between women who ended the treatment (n=42) and those who did not (n=50):** the comparison showed a significant difference only for phobic anxiety (*U*=-2.271, *p*=.023).

## DISCUSSION

The present study aimed to compare the cognitive and emotional performance of a sample of severely obese women that underwent the CT with a sample that had an SG for weight loss, taking into account the presence or absence of OSAS and comparing the results of both groups against controls.

Extended wait times for the necessary procedures (routine exams, night polysomnography, and consultations) may have impacted patients’ attendance and contributed to the reduced number of patients who reached the end of the study. For the purpose of surveillance and treatment of obesity and its comorbidities and preventing the associated risk of death, future studies should explore patients’ attendance data and reasons for attrition to provide better insights into their motives.

Data related to CR in the clinical samples emerged as extremely important because more than half of the patients had a low CR. The lowest CR was found in the oldest patients, in those with greater anthropometric measurements (e.g., neck circumference), and those with a higher burden of HTN, lowest cognitive performance and poorest levels of physical activity. Together with other studies that set out to investigate the role of CR in obesity-related deficits, our findings reinforce that CR can mitigate the association between these deficits and cognitive decline^5,7,25^. According to the evidence, high CR in an obese patient seems to reduce his vulnerability to vascular inflammation, neuropathology, cognitive deterioration, and incidence of dementia^26^. Interventions that address adherence and offer strategies for improve self-control should be more incisive on patients who are older, less educated, and have lower professional status. These strategies can promote involvement in sharing responsibility for treatment and contribute to the reduction of risk factors like HTN and DM, which our results show to be highly associated with OSA.

Regarding both treatments, the enhancement of results within the groups revealed that SG most influenced anthropometric measurements, but both methods affected the cognitive and emotional domains. An analysis of cognitive performance demonstrated that CT was relevant to the increase of attention, memory and interference, and SG was important for attention, memory and cognitive flexibility systems.

In our view, treatments may have induced the reaction of distinct neural circuits related to interference/inhibitory control (according to results from the Stroop test), speed of information processing and cognitive flexibility (from the results of TMT B and WCST), executive functions with a fundamental role in settings goals and objectives.

Inhibitory control contributes to the regulation of motivation to consume desirable food in response to environmental cues and speed of information processing. Cognitive flexibility implies the capacity to alternate one’s course of action and thoughts by promoting the process of adherence to new nutritional guidelines^27,28^. EF is also relevant for the attentional system and adjustment of behaviours, which are described as impoverished in the obese population due to decreased ability to influence emotional regulation.

EF components are thus important for successful outcomes^4^. Their improvement may be predominantly linked to an intensification of proactive inhibition of automatic or dominant behaviours during CT through its nutritional and psychological behavioural strategies. In the same way, SG may promote the restructuring of cognitive flexibility through changes in the stomach anatomy that require adaptive dietary modifications during the six months of follow-up, such as eating slowly, chewing food thoroughly, and increasing the frequency of meals^29^. It would be interesting to explore the maintenance of behaviours related to both interventions in a long-term follow-up.

In another regard, the decline in learning for both groups may reflect difficulty with the use of acquired information that favours changing behaviours. This can be a reflex of a passive learning style that underlines a general emotional difficulty. Together with the decrease of visuoperception, indicators suggest a poor understanding of the environment, low body awareness, and low body recognition. These obstacles seem to persist even 6 months after the interventions. To assure better apprehension of information and knowledge effectiveness, actions must fundamentally target health education and strategic behavioural changes. Cognitive training may also be an important interventional strategy.

The decrease of weight obtained through the CT, as well as the range of anthropometric measurements warranted by BS, are described as reasons for significant improvements to, or remission of, cardiovascular disease risk factors including HTN, DM, hyperlipidaemia, and OSA^30^. Their diminished prevalence, irrespective of the type of intervention, results in better anti-inflammatory state, better glucose metabolism and hypoxemic reverse, and may explain the incidence of cognitive and emotional improvement in OSA patients, particularly those who had a SG^31^.

No differences in cognitive scores emerged between the CT and SG groups after treatment. Some recent studies suggest that beneficial changes in cerebral structure and neural networks are promoted by rapid weight loss and might translate to cognitive changes over longer follow-up periods^24,32^. A finer analysis, however, evidences the positive value of weight loss in the treatment of these patients. Although the amount of weight loss achieved varies, better cognitive results between OSA groups reinforces evidence pointing to structural brain change namely an increase of gray matter volume in the hippocampus and frontal brain regions of OSA patients after treatment^33^. It will be relevant in future studies to verify the importance of OSA as related to the adherence to a weight loss treatment.

We found significant associations between emotional performance and the mean %WL following treatment. Considering that, SG evidenced a higher mean %WL and, compared with CT, a visible intragroup reduction of distress symptoms, we may infer that surgery was the most successful method for alleviating these dysfunctional symptoms. It is possible that individuals with higher weight loss developed a more adaptative approach to dealing with negative feelings, finding it easier to share and communicate these feelings, and hence increase mental well-being^34^. From our empirical evidence, weight status, vitality, and physical well-being are important tools in mental health and quality of life in the immediate post-surgery period.

Although emotional discomfort is associated with both obesity and OSA, it seems that the interventions had different effects on its expression, especially regarding anxiety and depressive symptoms. The reduction of these symptoms may be explained by the direct consequence of sleep improvement (e.g., reduction of insomnia, daytime sleepiness, fatigue, and snoring) or as a secondary result of the social effects that accompany resolution of both conditions^35^.

Patients that ended treatment differed from patients that did not only with respect to phobic anxiety. This kind of symptom implies a personal vulnerability possibly associated with the fear and disappointment of maintaining obesity and its comorbidities. Patients may reactively adopt avoidance behaviours regarding specific places or situations. Phobic anxiety may have functioned as a trigger that halted continuing treatment and efforts to acquire a healthy life.

Finally, the comparison of the CT sample with the control sample demonstrates the positive influence of weight loss on the speed of information processing and understanding the environment and a decrease of somatisation. However, CT did not benefit recent memory, which is responsible for the recall of meals, the increase of satiety process, the control of hunger, and food intake^36^. SG patients exhibited more perseverative responses and perseverative errors than controls, highlighting the maintenance of low cognitive flexibility and the incidence of these behaviours prior to surgery. Despite the weight loss, it is necessary that the intervention models improve autonomous regulation and offer a better perception of skills and the enrichment of self-control strategies that promote commitment to long-term behavioural changes.

Limitations regarding the representativeness of the studied population include our small sample size and the limitation to women. Although almost half of the patients with OSA develop depression at some stage, we evaluated but we did not exclude patients with depressive symptoms. We tried to understand if, according to the treatment, depressive symptoms decreased, and it happened for both groups of women with OSA. Methodological problems have also been identified regarding the absence of control in adhering to an eventual treatment for OSA with CPAP following polysomnography. However, we consider that the probability was equal for both groups that performed SG and CT.

The sample size may explain the absence of more important cognitive differences for patients after both interventions, and with a larger sample, maybe these differences could be more extensive. However, our predominantly female sample is similar to the typical candidates for the treatment of severe obesity in Portugal. A strength of this study is the longitudinal design and the inclusion of patients that performed a pre-treatment PSG.

## CONCLUSION

The findings suggest that when compared with CT, surgery for obesity positively influences a wide range of anthropometric, cognitive, and emotional domains, particularly in women with OSA. However, compared to patients in the control groups, the permanence of cognitive susceptibilities like low immediate memory and low cognitive flexibility may exercise influence on autonomous regulation and weight regain. The effects of additional weight gain, particularly those regarding the reversion of OSA mechanisms, must be addressed through longitudinal studies.

Psychological and nutritional interventions that address adherence to the intervention requirements and offer strategies for the enrichment of self-control and commitment should be mostly incisive. Even a small variation in weight may be considered a success of the therapeutic approach in terms of comorbidities, impact on the quality of life, and individual positive perception. Phobic anxiety is one of the most important distress symptoms contributing to the continuation of treatment. However, aspects such as patient-attendance data and reasons for attrition need to be further explored.
